# Supplementary material for: Interrogations of single-cell RNA splicing landscapes with SCASL define new cell identities with physiological relevance
Source: Nat Commun. 2024 Mar 9;15:2164. doi: 10.1038/s41467-024-46480-9 (PMC10925056; doi:10.1038/s41467-024-46480-9)
Supplement: Supplementary file 1 — Supplementary Information [file 41467_2024_46480_MOESM1_ESM.pdf]

Supplementary Figures

Interrogations of single-cell RNA splicing landscapes with SCASL define new cell identities with physiological relevance

Xianke Xiang[1,2], Yao He[3], Zemin Zhang[3,4,5], Xuerui Yang[1,2*]

[1] MOE Key Laboratory of Bioinformatics, School of Life Sciences, Tsinghua University, Beijing 100084, China

[2] Center for Synthetic & Systems Biology, Tsinghua University, Beijing 100084, China

[3] Biomedical Pioneering Innovation Center and School of Life Sciences, Peking-Tsinghua Center for Life Sciences, Academy for Advanced Interdisciplinary Studies, Peking University, Beijing 100871, China

[4] Changping Laboratory, Beijing 102206, China

[5] Cancer Research Institute, Shenzhen Bay Lab, Shenzhen 518132, China

*Correspondence: Xuerui Yang, School of Life Sciences, Tsinghua University, Beijing 100084, China. Tel: 86-10-62783943. Email: yangxuerui@tsinghua.edu.cn

Figure S1

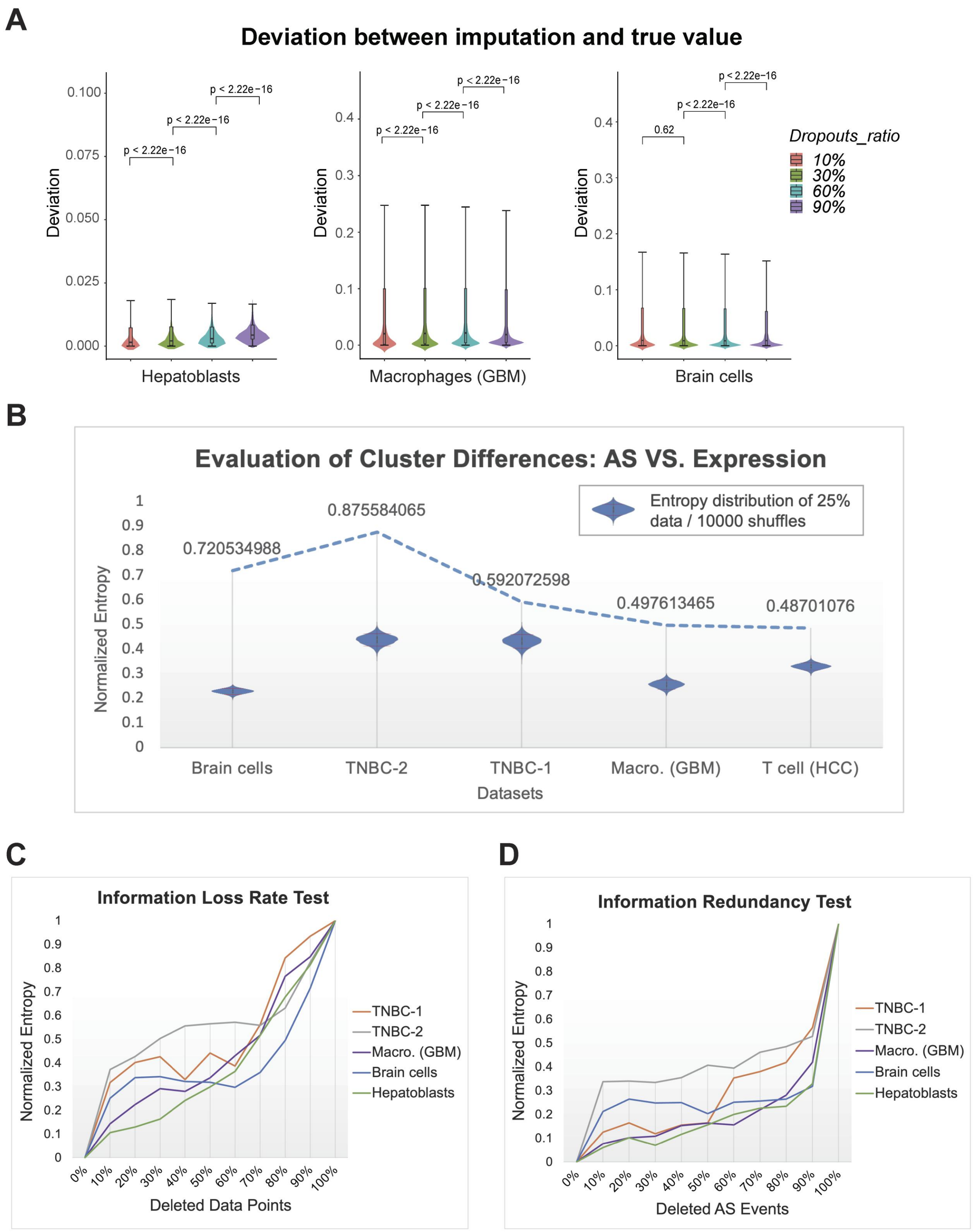
A
Deviation between imputation and true value
Deviation
Hepatoblasts
Macrophages (GBM)
Brain cells
p < 2.22e−16
0.62
Dropouts_ratio
10%
30%
60%
90%
B
Evaluation of Cluster Differences: AS VS. Expression
Entropy distribution of 25% data / 10000 shuffles
Normalized Entropy
0.720534988
0.875584065
0.592072598
0.497613465
0.48701076
Brain cells
TNBC-2
TNBC-1
Macro. (GBM)
T cell (HCC)
Datasets
C
Information Loss Rate Test
Normalized Entropy
Deleted Data Points
TNBC-1
TNBC-2
Macro. (GBM)
Brain cells
Hepatoblasts
D
Information Redundancy Test
Normalized Entropy
Deleted AS Events

Figure S1. Performance of SCASL.

(A) Violin plots showing the difference between the true and imputed AS probability values in 4 datasets with different percentages of the original data being dropped-out. Source data are provided as a Source Data file.

(B) The dashed line shows the normalized entropy between the clustering results generate from gene expression profiles (with Seurat) and AS profiles (with SCASL). The larger the entropy, the greater the difference between the clustering results. The violin plots show the distribution of the entropy calculated by comparing the cell cluster labels obtained from the gene expression data vs. the labels of which 25% were randomly shuffled 10,000 times.

(C) Different percentages of the non-NA values were randomly selected and dropped out. The lines show the entropies between the clustering results by SCASL based on the original data and the data with forced data drop-outs.

(D) Different percentages of the AS sites were randomly removed. The lines show the entropies between the clustering results by SCASL based on the original data and the data with AS sites removed.

Figure S2

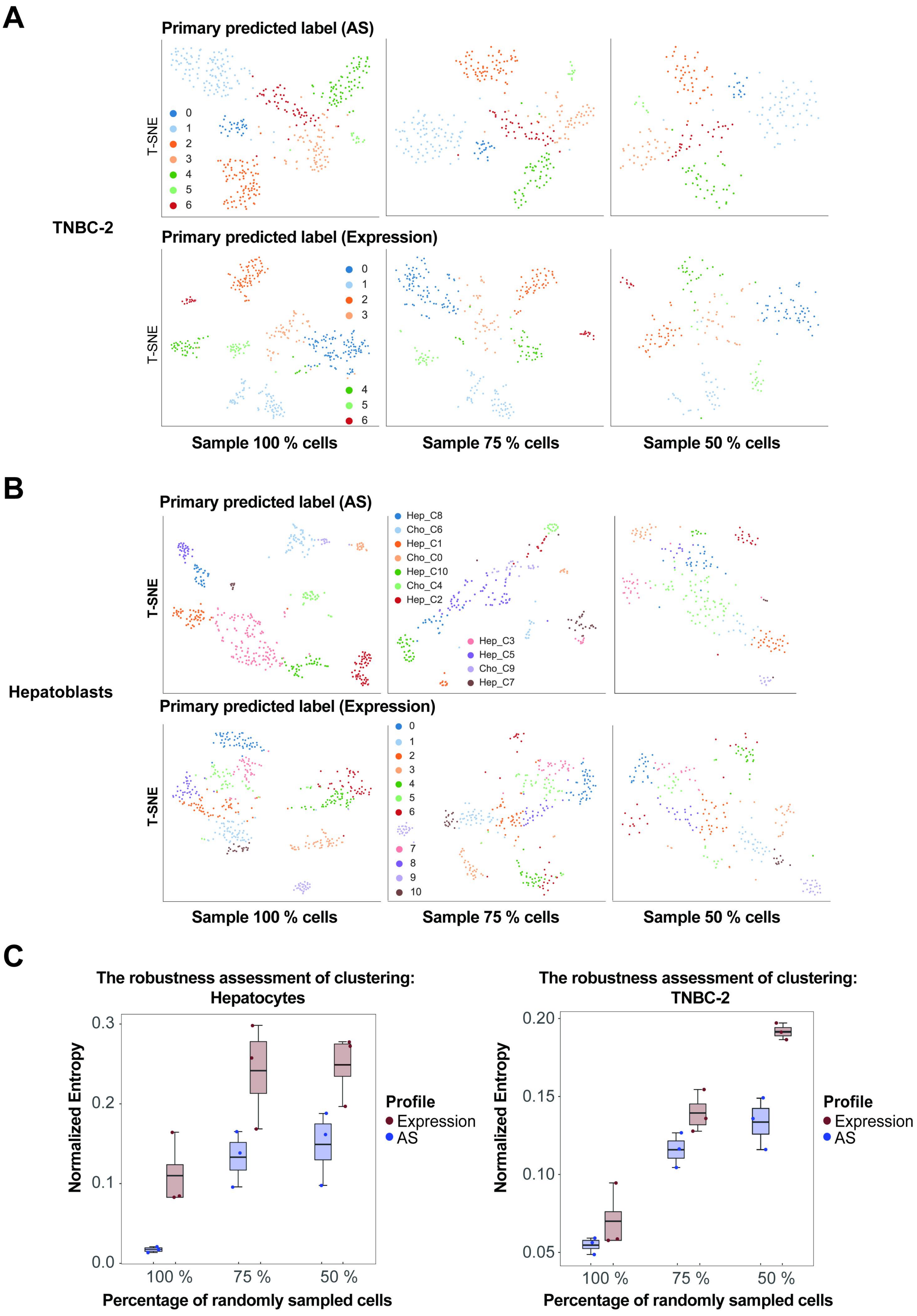
A
TNBC-2
Primary predicted label (AS)
Primary predicted label (Expression)
T-SNE
0
1
2
3
4
5
6
Sample 100 % cells
Sample 75 % cells
Sample 50 % cells
B
Hepatoblasts
Primary predicted label (AS)
Hep_C8
Cho_C6
Hep_C1
Cho_C0
Hep_C10
Cho_C4
Hep_C2
Hep_C3
Hep_C5
Cho_C9
Hep_C7
Primary predicted label (Expression)
7
8
9
10
C
The robustness assessment of clustering: Hepatocytes
The robustness assessment of clustering: TNBC-2
Normalized Entropy
0.0
0.1
0.2
0.3
0.05
0.10
0.15
0.20
100 %
75 %
50 %
Percentage of randomly sampled cells
Profile
Expression
AS

Figure S2. Robustness assessment of SCASL.

(A, B) Representative visualization results were selected from the clustering analysis of the TNBC-2 dataset (A) and hepatoblasts dataset (B) by sampling 100%, 75%, and 50% of the cells. The upper row displays the clustering results of the AS profile, while the lower row shows the clustering results of the expression profile.
(C) The box plot shows the comparison of each clustering result with the original clustering result when 100%, 75%, and 50% of cells were randomly sampled multiple times in the hepatoblast data set and TNBC-2 data set. The AS profiles were used by SCASL and the gene expression profiles were used by Seurat for the clustering analyses.

Figure S3

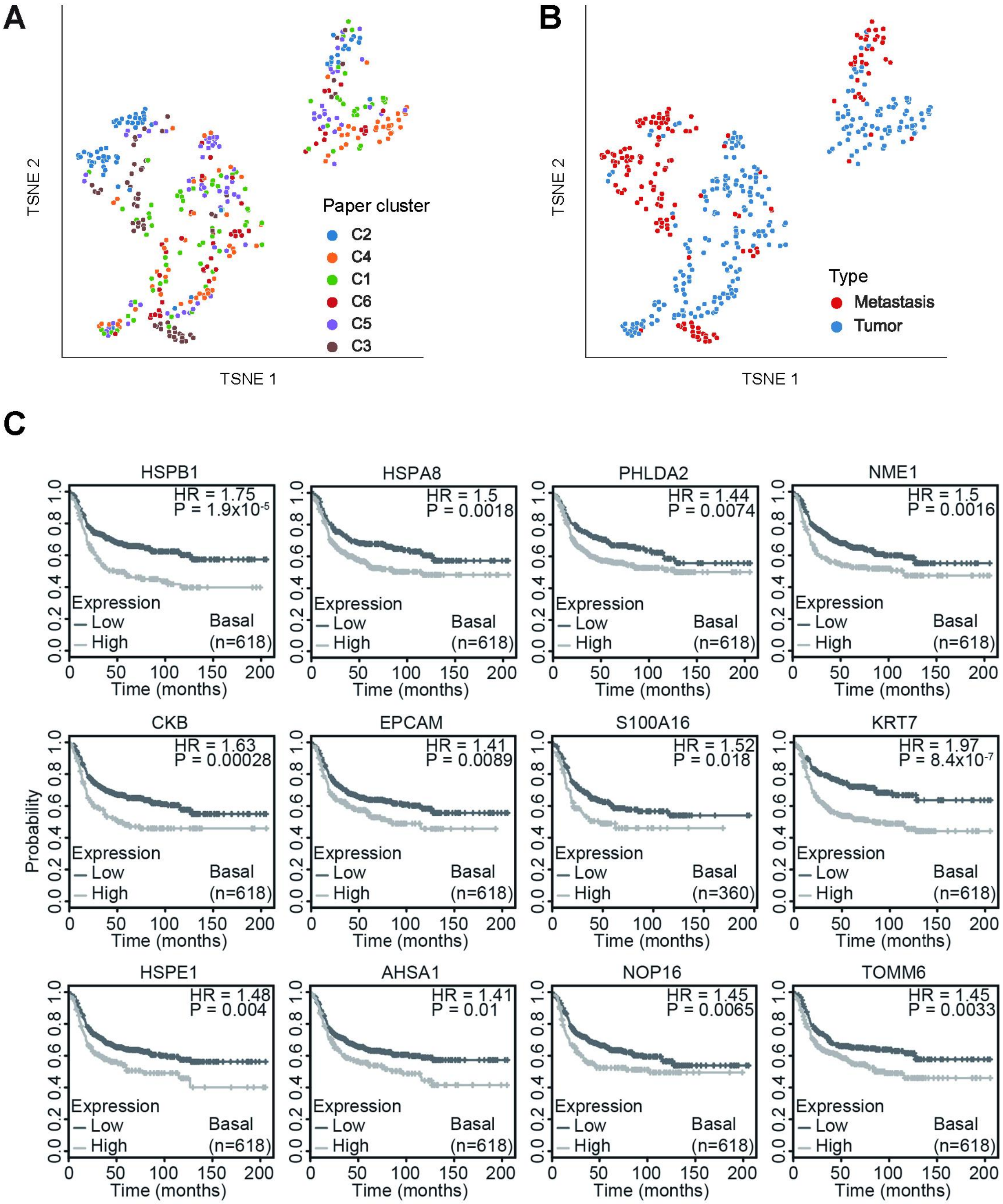

Figure S3. Clustering results of the micrometastasis TNBC data.

(A, B) UMAP plot showing clustering of 422 TNBC tumor cells by SCASL based on the AS landscapes. The cells are color labeled by the clusters defined in the original article of data resource (A) or by the tissue source, i.e., primary tumor or micrometastasis (B). **(C)** Survival analysis of the breast cancer patients in TCGA partitioned by the signature genes of cluster 0.

Figure S4

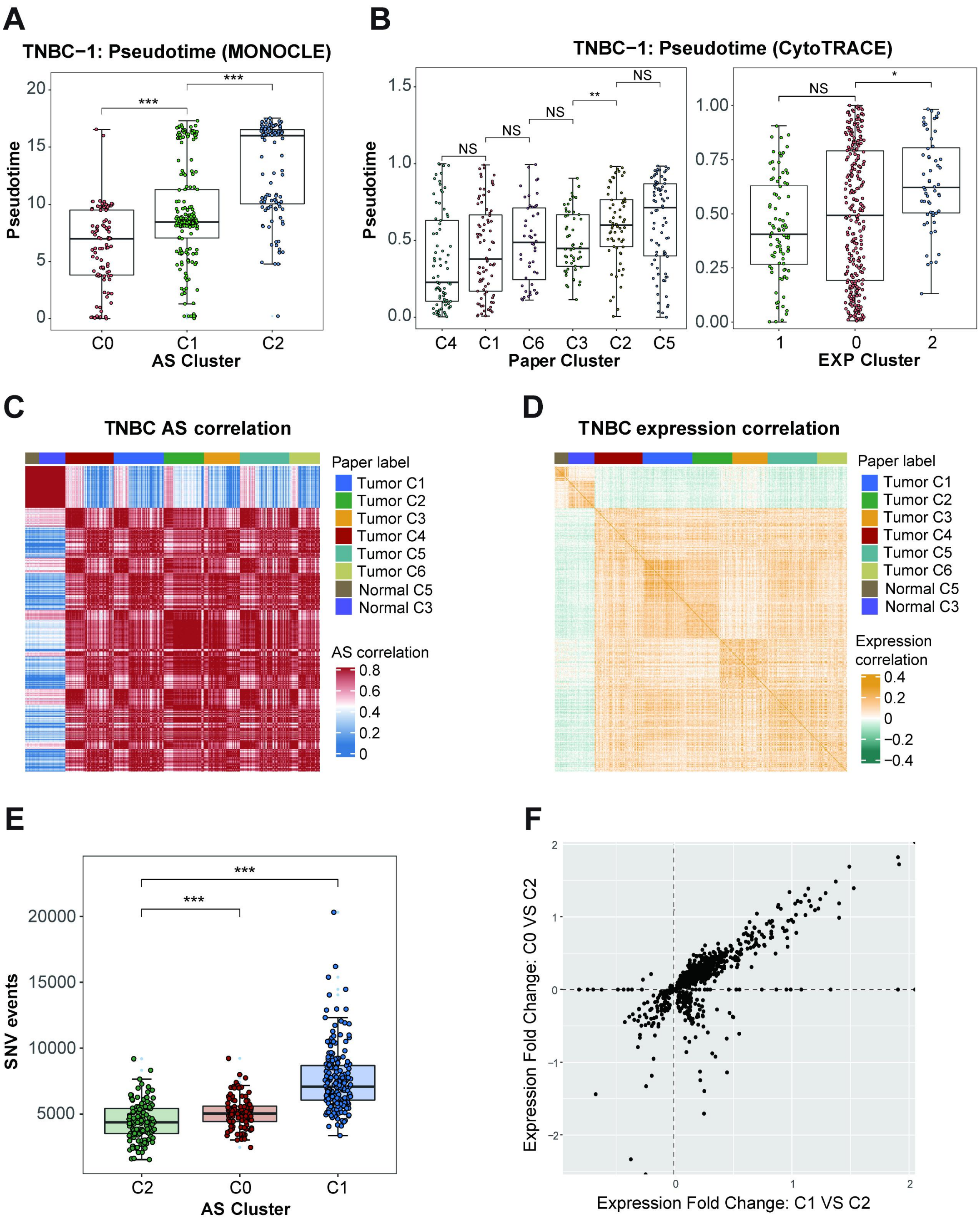
A
TNBC−1: Pseudotime (MONOCLE)
Pseudotime
AS Cluster
C0
C1
C2
B
TNBC−1: Pseudotime (CytoTRACE)
Paper Cluster
C4
C1
C6
C3
C2
C5
EXP Cluster
NS
C
TNBC AS correlation
Paper label
Tumor C1
Tumor C2
Tumor C3
Tumor C4
Tumor C5
Tumor C6
Normal C5
Normal C3
AS correlation
D
TNBC expression correlation
Expression correlation
E
SNV events
AS Cluster
F
Expression Fold Change: C0 VS C2
Expression Fold Change: C1 VS C2

Figure S4. Analysis of the clustering results with the micrometastasis TNBC data.

(A) Pseudo-time analysis of the AS clustering results was performed using MONOCLE. Wilcoxon rank-sum test were used to evaluate the statistical significance between different groups (two-sided test, using Bonferroni correction to adjust for multiple comparisons). *: p-value <0.05; **: p-value <0.01, ***: p-value <0.001. Data are presented as median values +/- SEM. Each box shows the median and interquartile range (IQR 25th-75th percentiles). Exact p-values are provided as a Source Data file.
(B) Pseudo-time analysis of the gene expression clustering results was performed using CytoTRACE (supplementary to Fig. 2C). The clustering labels on the left side are derived from the original paper of the dataset, while the clustering labels on the right side correspond to the clustering results obtained from Seurat based on gene expression (with the same number of clusters as the AS clusters). Exact p-values are provided as a Source Data file.
(C, D) Heat map showing the spearman correlation coefficient between the AS profiles (C) or gene expression profiles (D) of the TNBC and normal epithelial cells, arranged by the clusters defined in the original article of data resource.
(E) Numbers of SNVs in the single-cells inferred using MonoVar. Exact p-values are provided as a Source Data file.
(F) Fold changes of differentially expressed genes by comparing C0 vs. C2 (Y-axis) and C1 vs. C2 (X-axis).

Figure S5

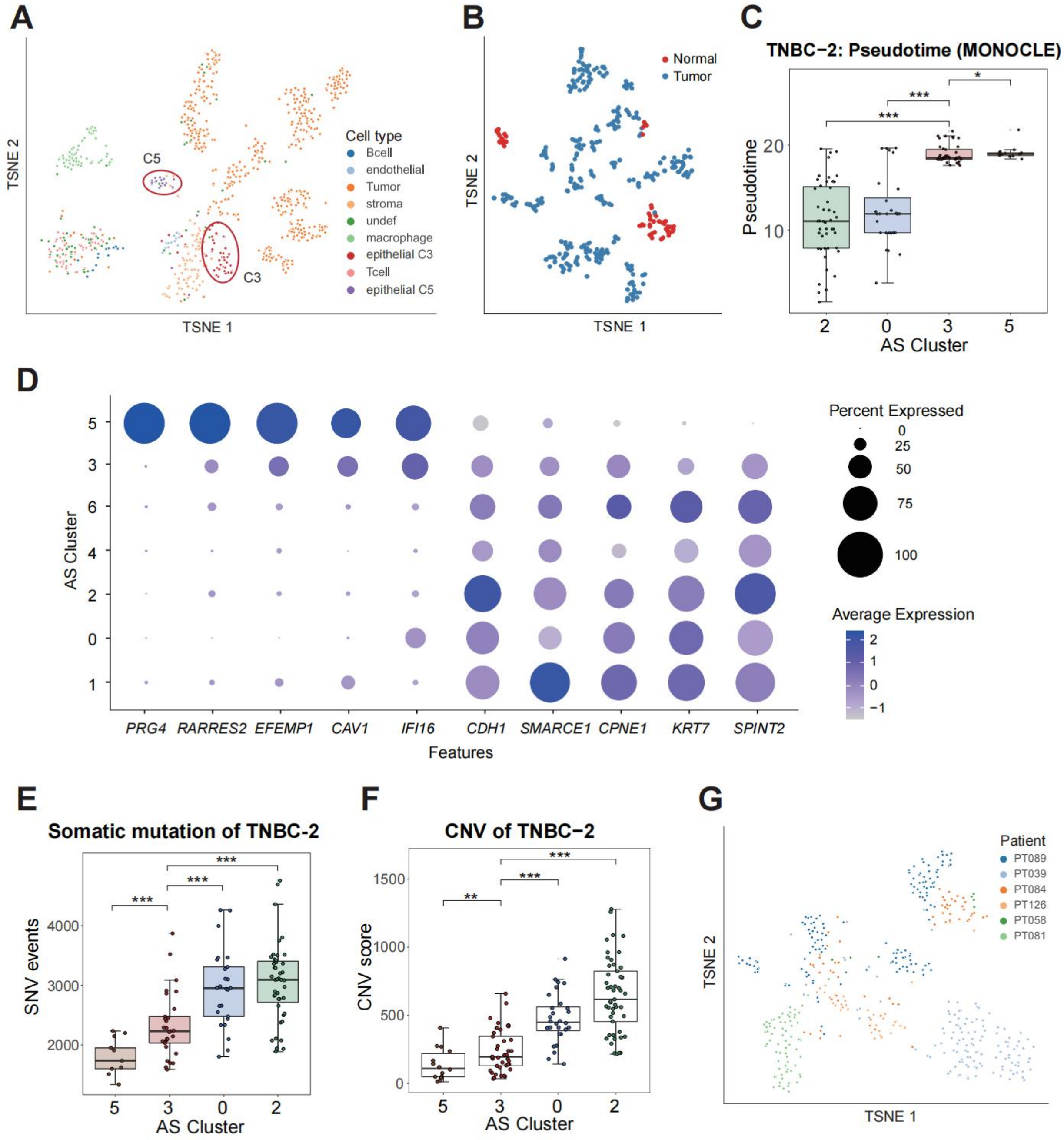

Figure S5. Analysis of the clustering results with the primary TNBC dataset.

(A) TSNE plot showing clustering of TNBC tumor cells and other non-cancerous cells based on AS landscapes.

(B) TSNE plot showing clustering of TNBC tumor cells based on gene expression profile.

(C) Pseudo-time analysis of the AS clustering results was performed using MONOCLE. T-tests were used to evaluate the statistical significance between different groups. *, <0.05; **, <0.01, ***, <0.001. Exact p-values are provided as a Source Data file.
(D) Expression levels of selected signature genes of C5 and C3.
(E) Numbers of SNVs in the single-cells inferred using MonoVar. Exact p-values are provided as a Source Data file.
(F) Copy number variation analysis of the single-cells was performed using inferCNV. Exact p-values are provided as a Source Data file.
(G) TSNE plot showing clustering of 443 normal epithelial cells and primary TNBC tumor cells based on AS landscapes. Different patients are marked on the plot.

Figure S6

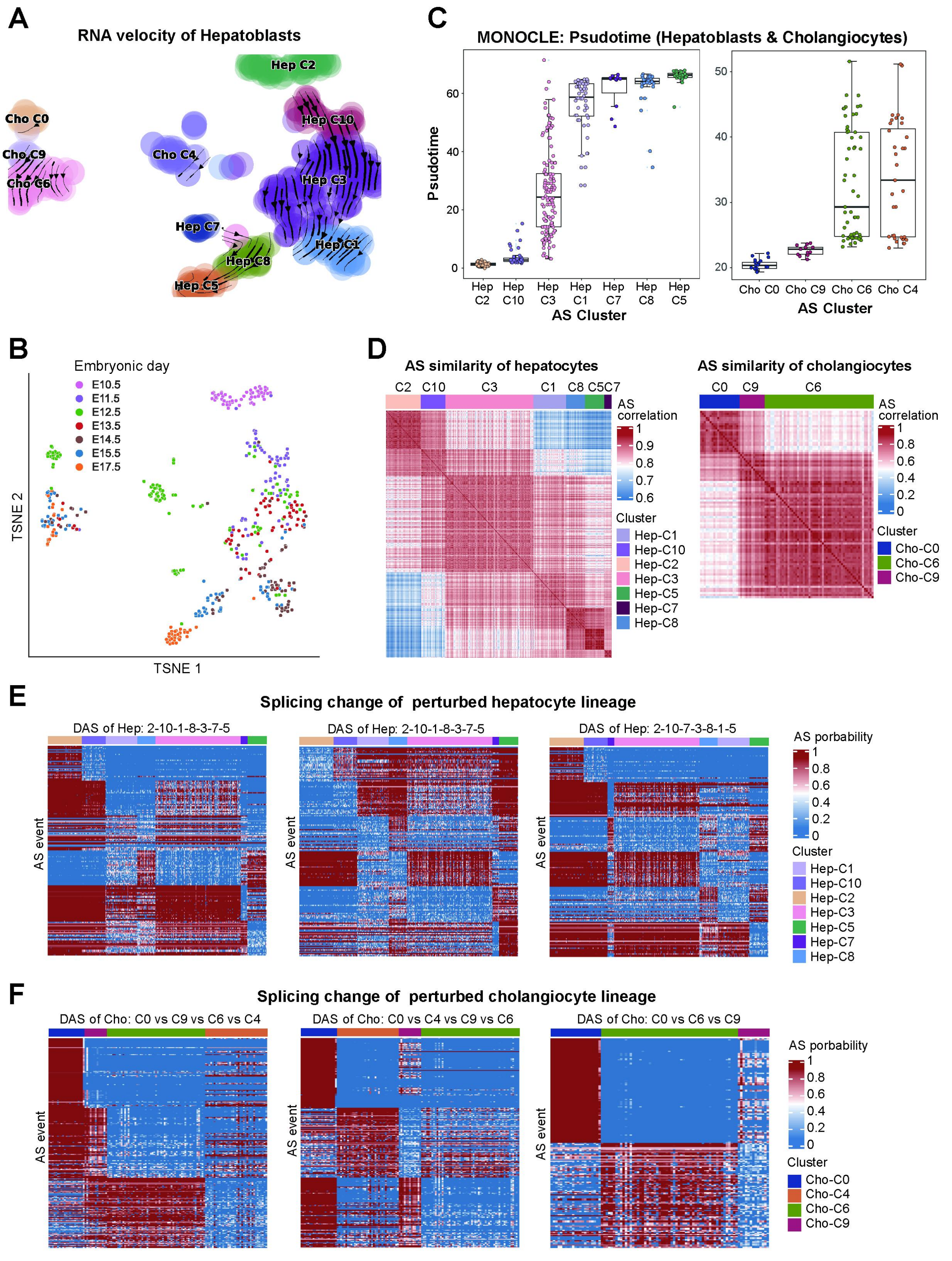
A
RNA velocity of Hepatoblasts
Hep C2
Hep C10
Cho C0
Cho C9
Cho C6
Cho C4
Hep C3
Hep C7
Hep C1
Hep C8
Hep C5
B
Embryonic day
E10.5
E11.5
E12.5
E13.5
E14.5
E15.5
E17.5
TSNE 2
TSNE 1
C
MONOCLE: Psudotime (Hepatoblasts & Cholangiocytes)
Psudotime
Hep C2
Hep C10
Hep C3
Hep C1
Hep C7
Hep C8
Hep C5
AS Cluster
Cho C0
Cho C9
Cho C6
Cho C4
AS Cluster
D
AS similarity of hepatocytes
C2 C10 C3 C1 C8 C5C7
AS correlation
Cluster
Hep-C1
Hep-C10
Hep-C2
Hep-C3
Hep-C5
Hep-C7
Hep-C8
AS similarity of cholangiocytes
C0 C9 C6
AS correlation
Cluster
Cho-C0
Cho-C6
Cho-C9
E
Splicing change of perturbed hepatocyte lineage
DAS of Hep: 2-10-1-8-3-7-5
DAS of Hep: 2-10-1-8-3-7-5
DAS of Hep: 2-10-7-3-8-1-5
AS event
AS porbability
Cluster
Hep-C1
Hep-C10
Hep-C2
Hep-C3
Hep-C5
Hep-C7
Hep-C8
F
Splicing change of perturbed cholangiocyte lineage
DAS of Cho: C0 vs C9 vs C6 vs C4
DAS of Cho: C0 vs C4 vs C9 vs C6
DAS of Cho: C0 vs C6 vs C9
AS event
AS porbability
Cluster
Cho-C0
Cho-C4
Cho-C6
Cho-C9

Figure S6. Analysis of the hepatoblast differentiation lineages.

(A) RNA velocity analysis of 447 embryonic liver cells clustered by SCASL based on AS. The arrows in RNA velocity indicate the direction and velocity of dynamic changes in gene expression.
(B) TSNE plot showing clustering of 447 embryonic liver cells by SCASL based on AS profiles. The cells are labeled by embryonic time.
(C) Pseudo-time analysis of the AS clustering results was performed using MONOCLE. On the left are clusters of cells belonging to hepatoblasts and hepatocytes, and on the right are clusters of cells belonging to cholangiocytes
(D) Heat maps showing the spearman correlation coefficient between the AS profiles of the clusters defined by SCASL, arranged by the hepatocyte lineage and cholangiocyte lineage.
(E, F) Supplementary to Fig. 4E, F. AS profiles of the top differential splicing events based on pairwise comparisons between the randomly shuffled clusters along the hepatocyte (E) and cholangiocyte (F) lineages.

Figure S7

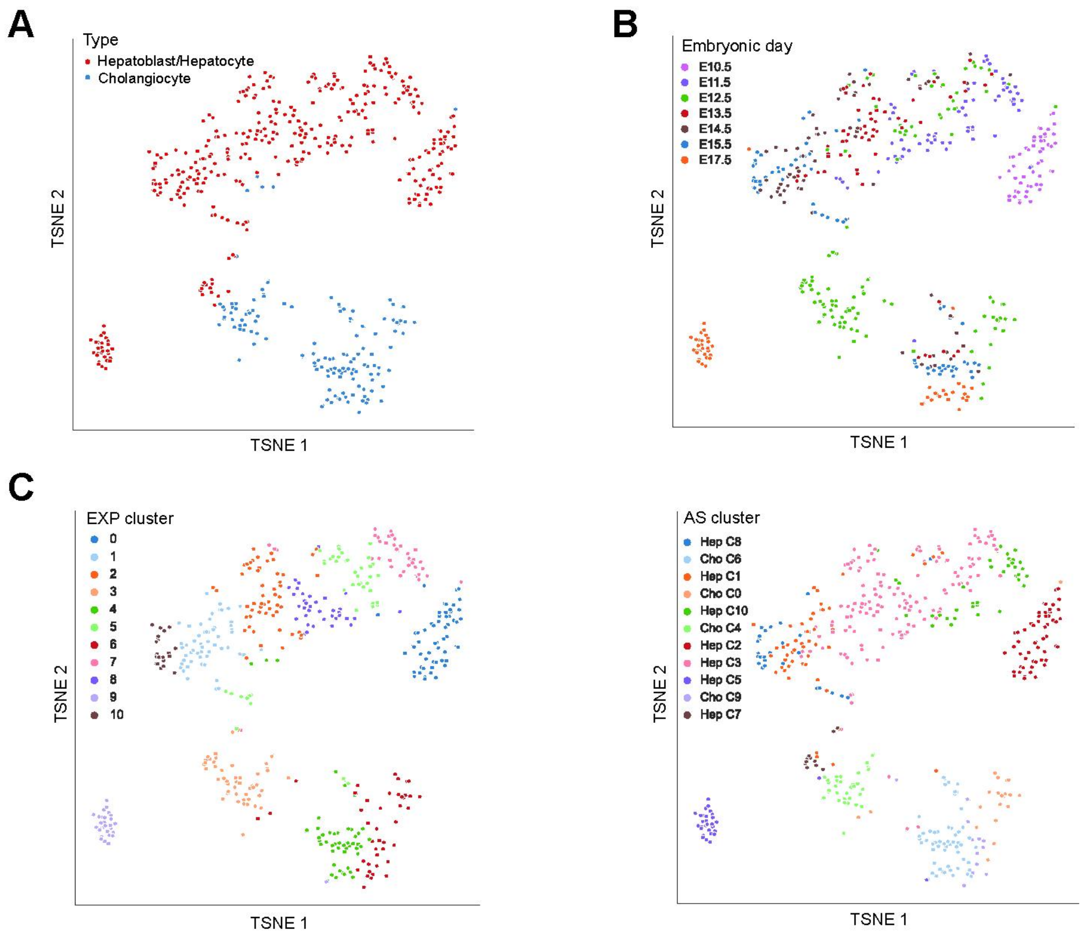

Figure S7. Clustering results based on gene expression of the hepatoblasts.

(A-C) TSNE plot showing clustering of 447 embryonic liver cells by Seurat based on expression profiles. Cells are labeled according to the cell type indicated in the original paper (A), embryonic time (B), clusters defined by Seurat (C, left), or clusters defined by SCASL based on AS (C, right).

Figure S8

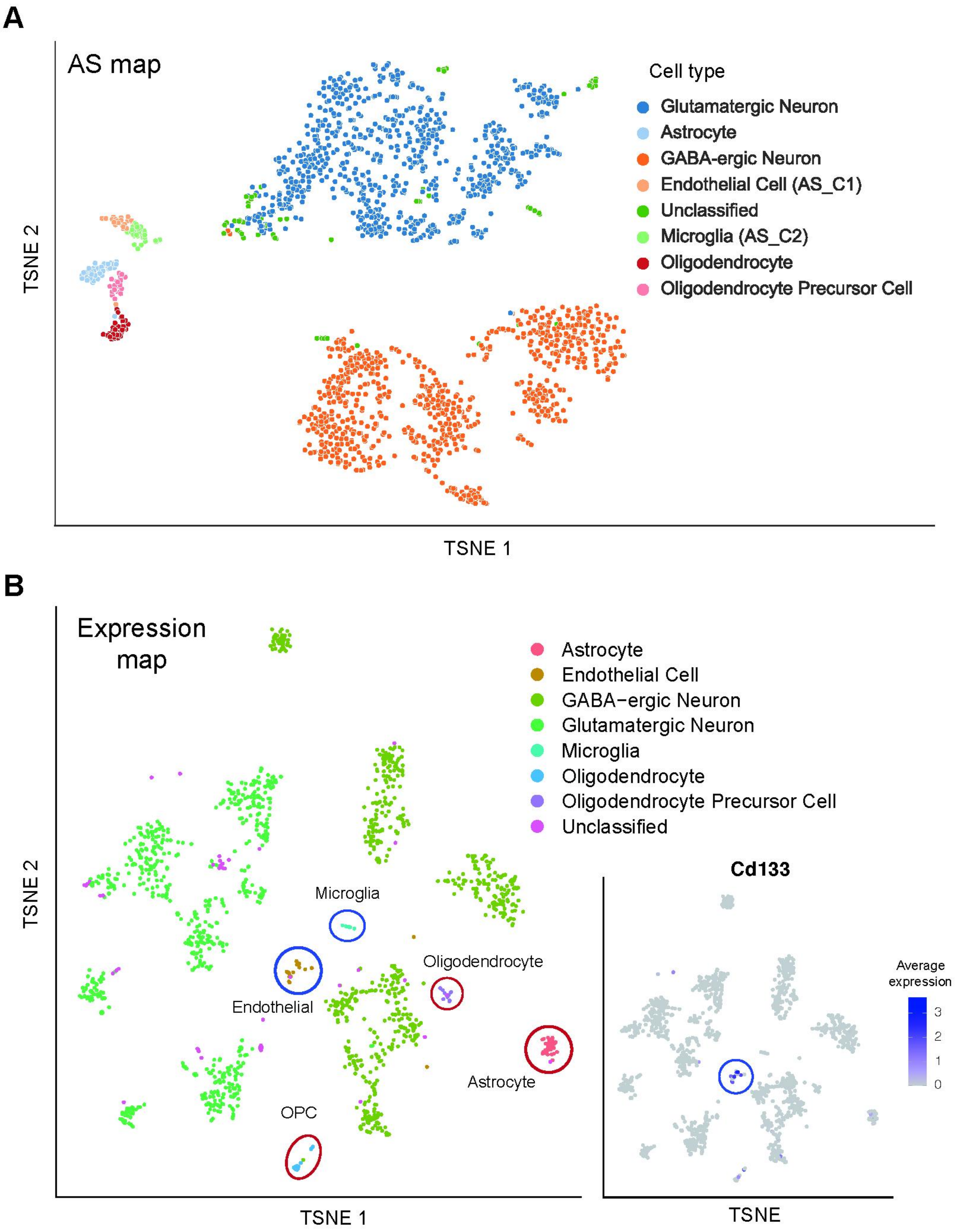
A
AS map
Cell type
Glutamatergic Neuron
Astrocyte
GABA-ergic Neuron
Endothelial Cell (AS_C1)
Unclassified
Microglia (AS_C2)
Oligodendrocyte
Oligodendrocyte Precursor Cell
TSNE 2
TSNE 1
B
Expression map
Astrocyte
Endothelial Cell
GABA−ergic Neuron
Glutamatergic Neuron
Microglia
Oligodendrocyte
Oligodendrocyte Precursor Cell
Unclassified
Microglia
Endothelial
Oligodendrocyte
Astrocyte
OPC
TSNE 2
TSNE 1
Cd133
Average expression
3
2
1
0
TSNE

Figure S8. Clustering results by SCASL based on the AS profiles of mouse brain cells.

(A) TSNE plot showing clustering of mouse brain cells by SCASL based on AS profiles. Cell types are labels on the plot.
(B) TSNE plot showing clustering of mouse brain cells by Seurat based on gene expression profiles. Cell types are labels on the plot. The figure to the right shows the expression level of endothelial progenitor cell (EPC) marker CD133.

Figure S9

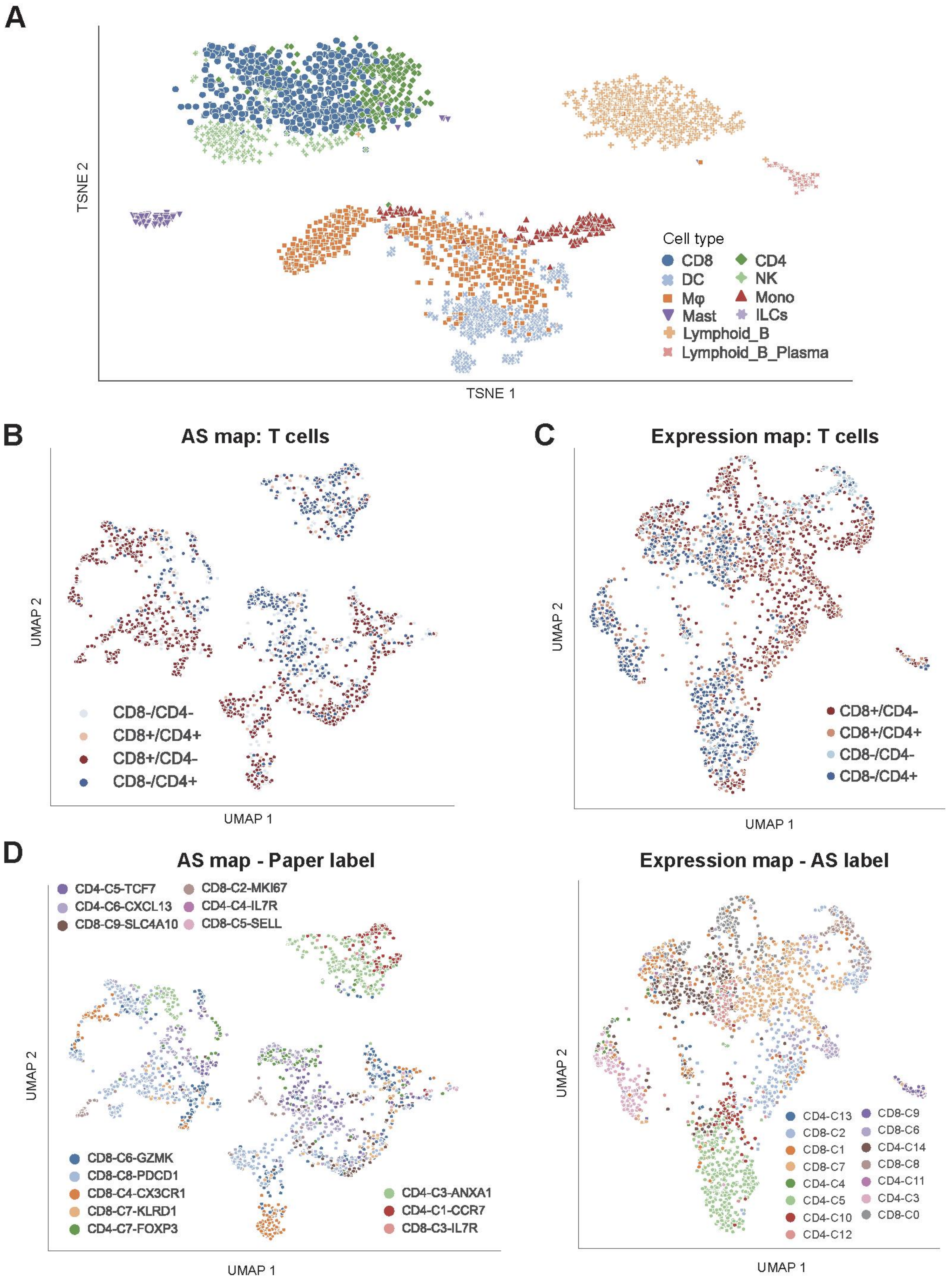

A
TSNE 2
TSNE 1
Cell type
CD8
CD4
DC
NK
Mφ
Mono
Mast
ILCs
Lymphoid_B
Lymphoid_B_Plasma
B
AS map: T cells
UMAP 2
UMAP 1
CD8-/CD4-
CD8+/CD4+
CD8+/CD4-
CD8-/CD4+
C
Expression map: T cells
UMAP 2
UMAP 1
CD8+/CD4-
CD8+/CD4+
CD8-/CD4-
CD8-/CD4+
D
AS map - Paper label
CD4-C5-TCF7
CD8-C2-MKI67
CD4-C6-CXCL13
CD4-C4-IL7R
CD8-C9-SLC4A10
CD8-C5-SELL
CD8-C6-GZMK
CD8-C8-PDCD1
CD8-C4-CX3CR1
CD8-C7-KLRD1
CD4-C7-FOXP3
CD4-C3-ANXA1
CD4-C1-CCR7
CD8-C3-IL7R
UMAP 2
UMAP 1
Expression map - AS label
CD4-C13
CD8-C9
CD8-C2
CD8-C6
CD8-C1
CD4-C14
CD8-C7
CD8-C8
CD4-C4
CD4-C11
CD4-C5
CD4-C3
CD4-C10
CD8-C0
CD4-C12
UMAP 2
UMAP 1

Figure S9. Clustering of HCC tumor-associated immune cells.

(A) UMAP plot showing clustering of the HCC tumor-associated immune cells by SCASL based on AS profiles. The principal component analysis (PCA) is performed with a dimensionality reduction number of 30.
(B, C) UMAP plot showing clustering of 2349 T cells by SCASL based on AS profiles (B) or by Seurat based on gene expression profiles with 4000 variable features (C). Cells are labeled based on expressions of CD4 and CD8. The PCA is performed with a dimensionality reduction number of 30 for both methods.
(D) UMAP plot showing clustering of 2349 T cells by SCASL based on AS profiles (left) or by Seurat based on gene expression profiles (right). The cells are labeled by the clusters defined by Seurat with gene expression data (left) or by SCASL with AS profiles (right).

Figure S10

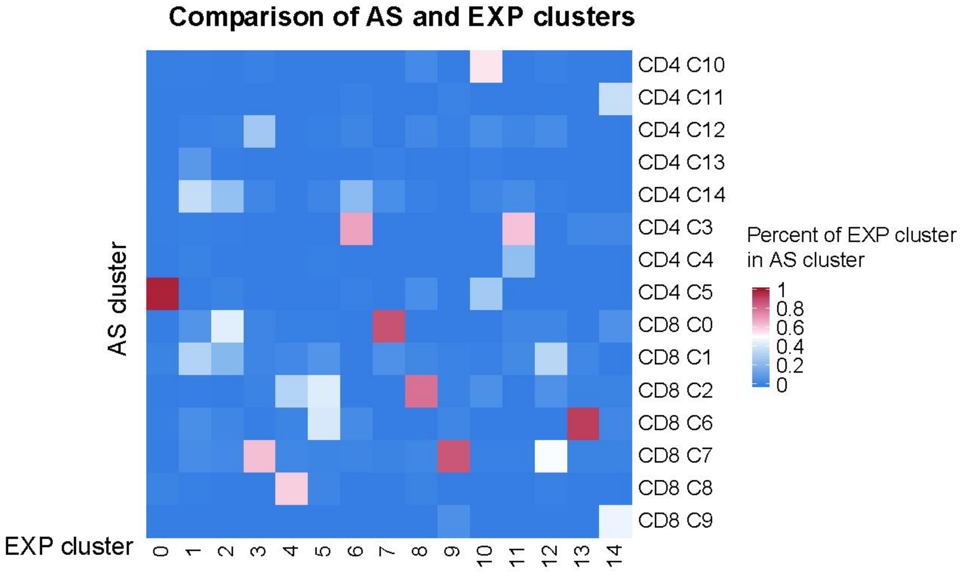

Figure S10. Comparison of the clusters defined by AS or expression of the T cells.

Heatmap showing overlaps between the clusters defined based on AS or gene expression data of the T cells. The cell clusters defined by Seurat based on gene expression are arranged by columns, whereas the clusters defined by SCASL based on AS are arranged by rows. The color indicates the proportions of the overlapping cells in the expression clusters.

Figure S11

A

Supplementary to Fig. 5D

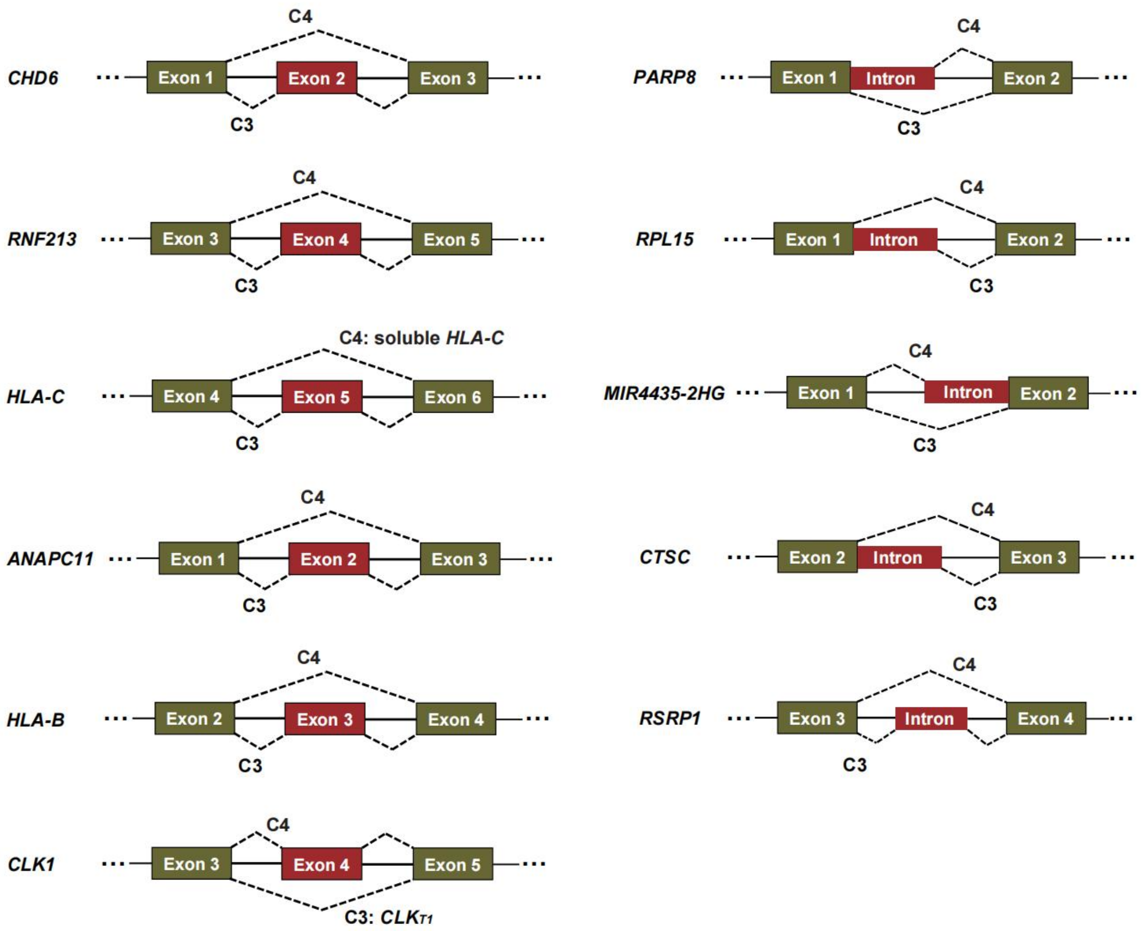

B

Corresponding to Fig. 5C, Fig. 6B,D

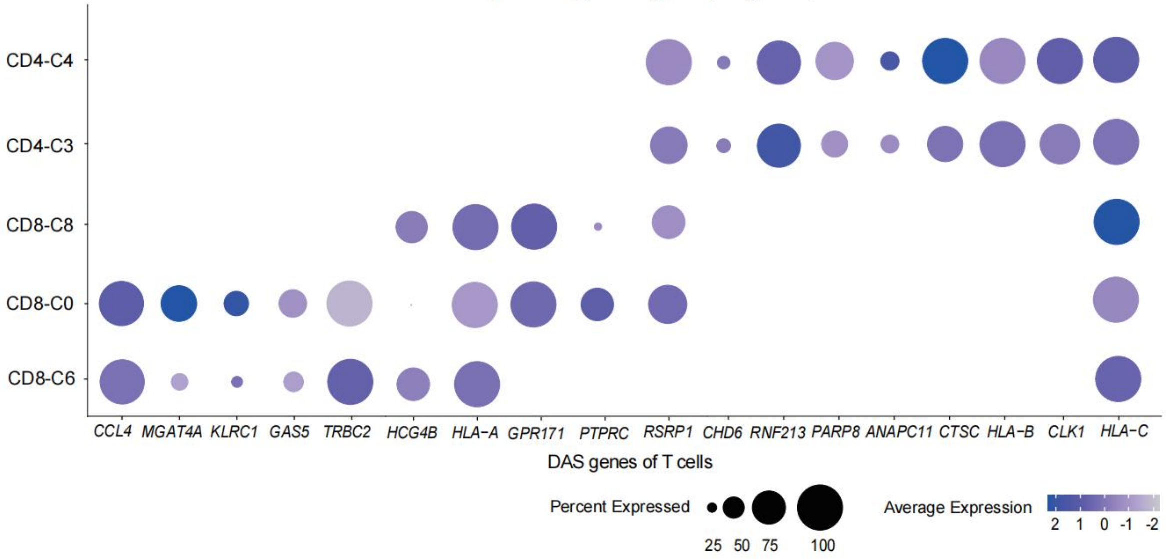

Figure S11. Supplementary details for Figures 5 and 6.

(A) Schematic representation of differential splicing events in C3 and C4 shown in Fig. 5C.

(B) Expression levels of the genes with significantly differential splicing listed in Figs. 5C, 6B, C.

Figure S12

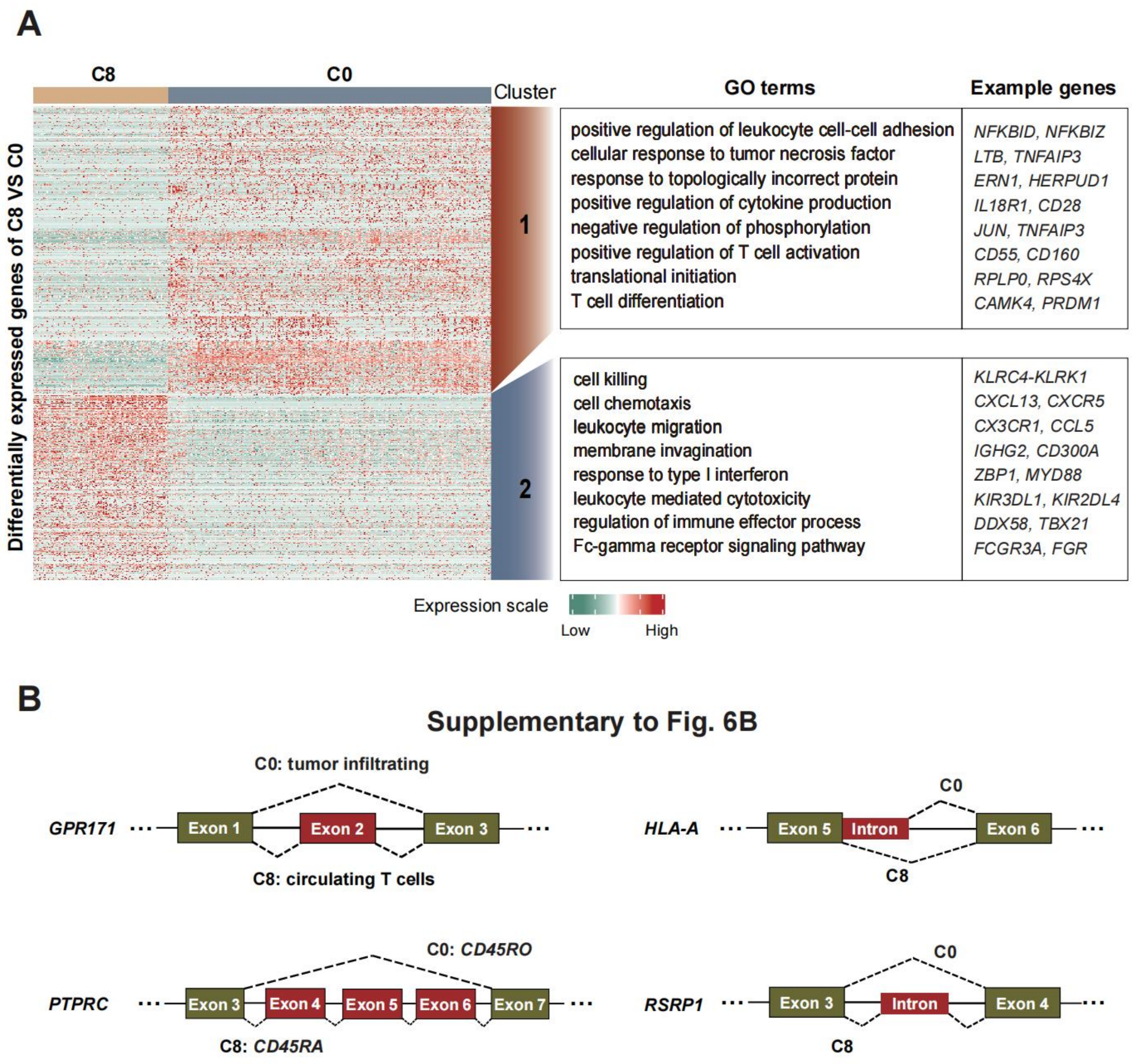

Figure S12. Comparisons between CD8 T cells in C0 and C8.

(A) Heatmap showing the differentially expressed genes (P-value < 1e-5) in C0 VS C8. The GO functional enrichment results of these genes are listed to the right, as well as some representative genes.

(B) Schematic representation of differential splicing events in C0 and C8 shown in Fig. 6B.

Figure S13

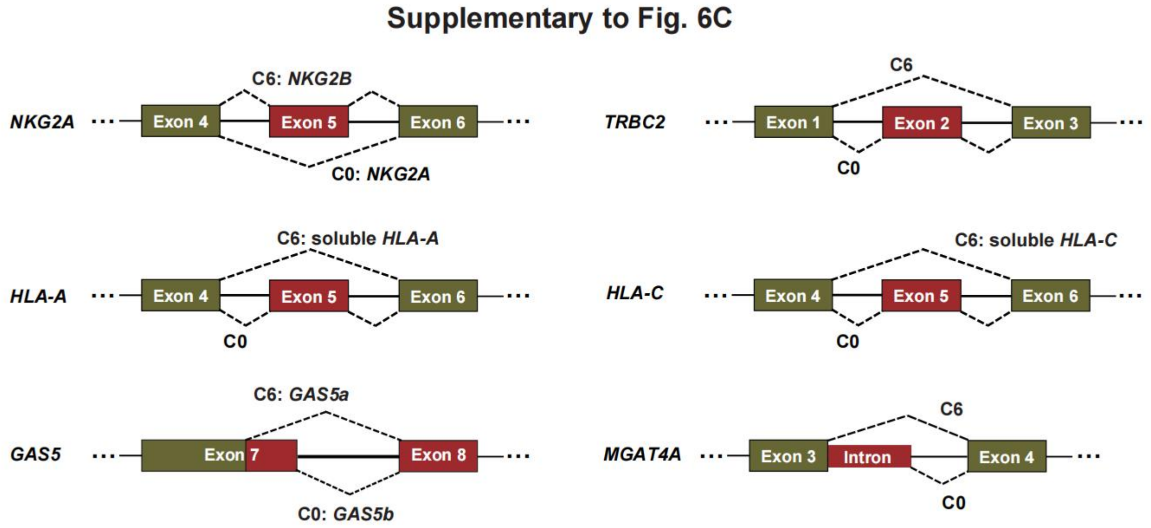

Figure S13. Schematic representations of differential splicing events.

Schematic representation of the differential splicing events in C6 vs. C0 shown in Fig. 6D.

Figure S14

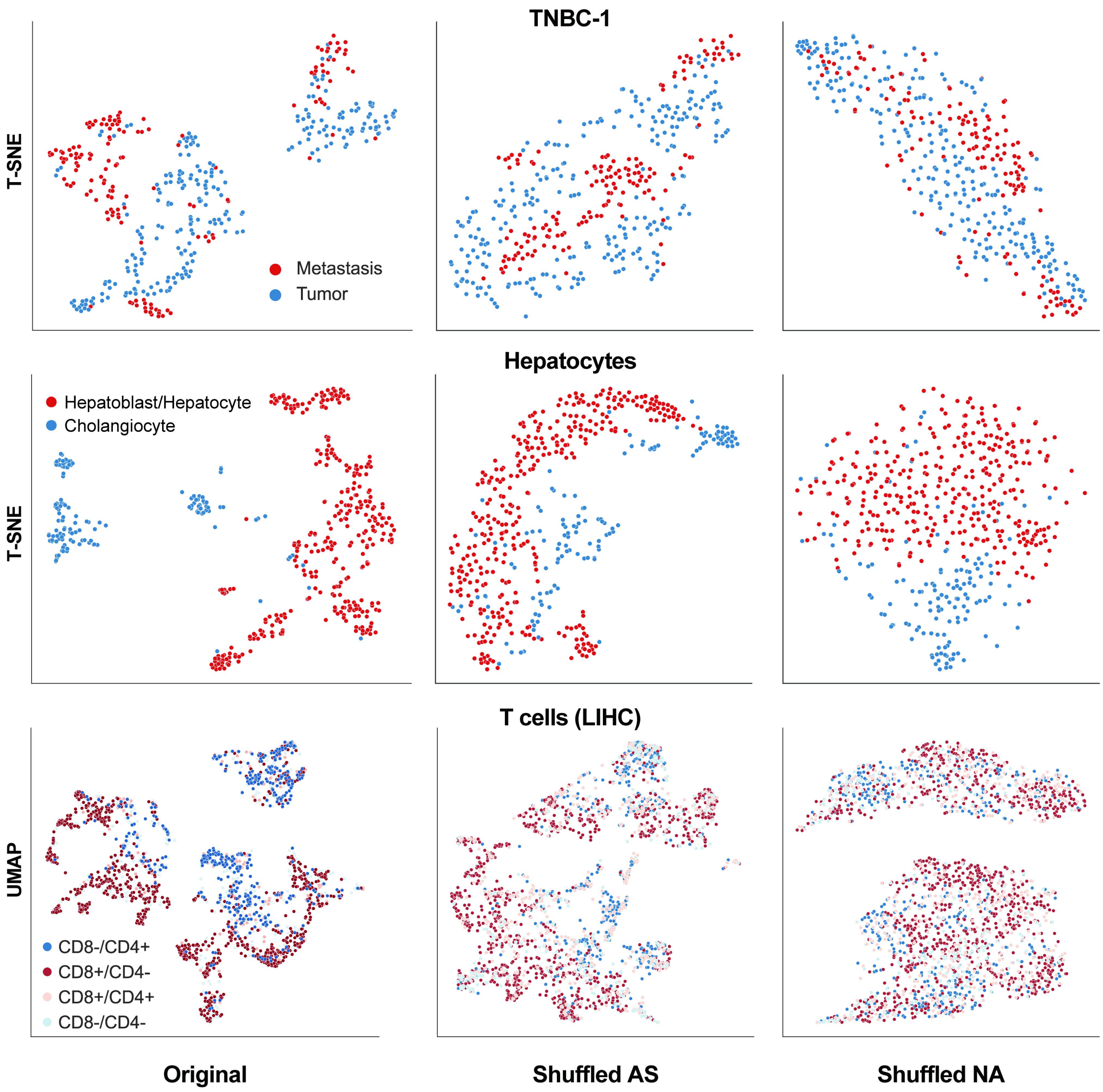

Figure S14. Clustering results with AS and missing data information.

Figures on the left show the clustering results using the complete process of SCASL (AS and NA information). Figures in the middle show the clusters defined by SCASL with the AS profile shuffled. Figures on the right show the clusters defined by SCASL with the missing data information shuffled.

Figure S15

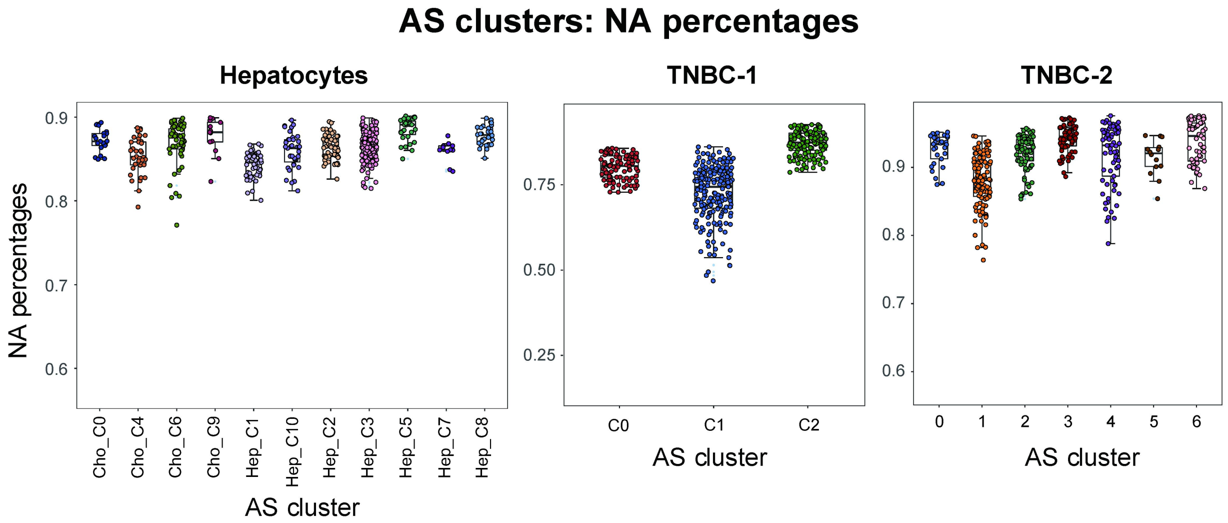

Figure S15. Comparison of NA percentages in different clusters.

The percentages of missing AS probability values (NA values) across the cell clusters defined by SCASL are shown in box plots.

Figure S16

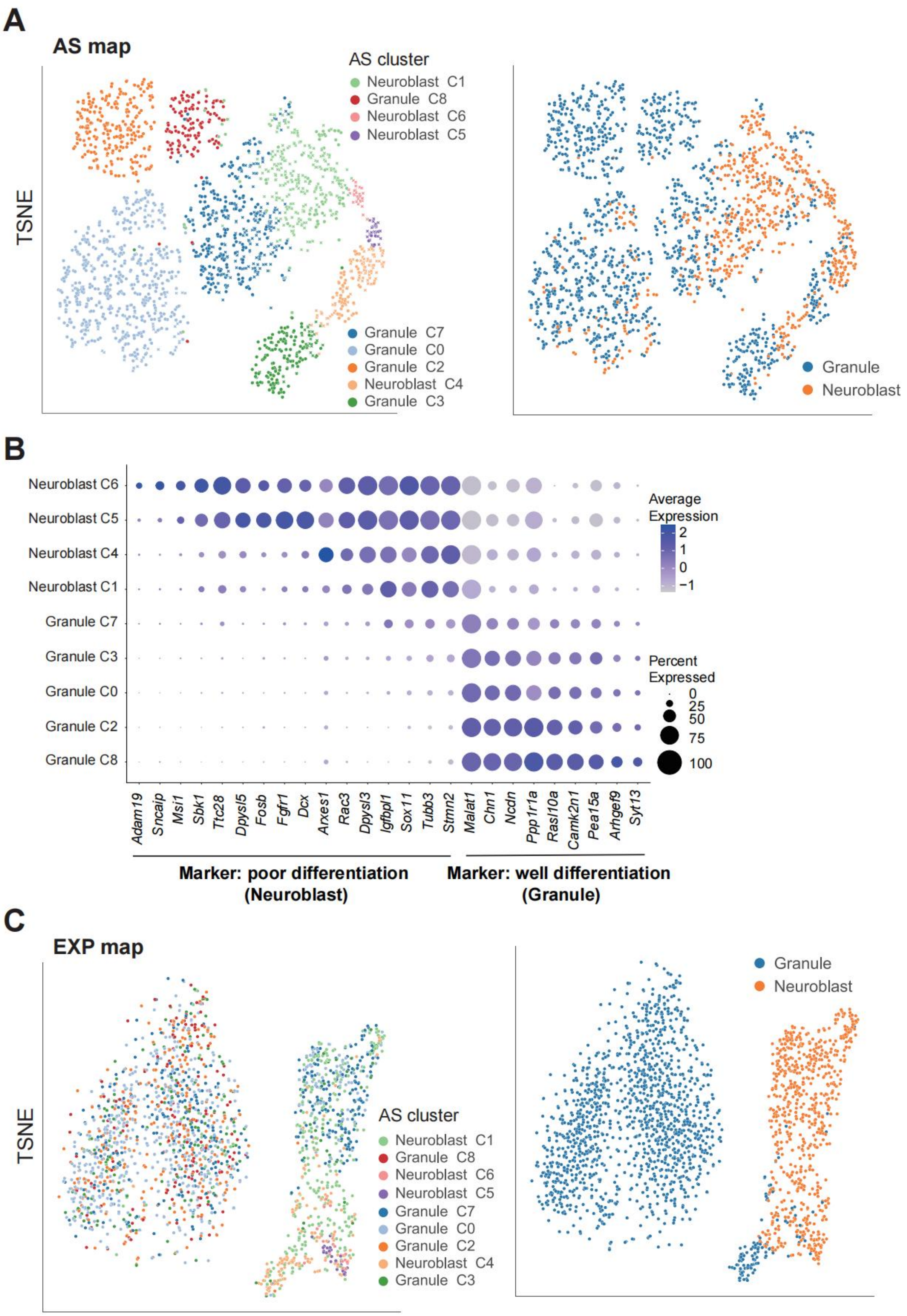
A
AS map
TSNE
AS cluster
Neuroblast C1
Granule C8
Neuroblast C6
Neuroblast C5
Granule C7
Granule C0
Granule C2
Neuroblast C4
Granule C3
Granule
Neuroblast
B
Neuroblast C6
Neuroblast C5
Neuroblast C4
Neuroblast C1
Granule C7
Granule C3
Granule C0
Granule C2
Granule C8
Average Expression
2
1
0
−1
Percent Expressed
0
25
50
75
100
Adam19
Sncaip
Msi1
Sbk1
Ttc28
Dpysl5
Fosb
Fgfr1
Dcx
Arxes1
Rac3
Dpysl3
Igfbpl1
Sox11
Tubb3
Stmn2
Malat1
Chn1
Ncdn
Ppp1r1a
Rasl10a
Camk2n1
Pea15a
Arhgef9
Syt13
Marker: poor differentiation (Neuroblast)
Marker: well differentiation (Granule)
C
EXP map
TSNE
AS cluster
Neuroblast C1
Granule C8
Neuroblast C6
Neuroblast C5
Granule C7
Granule C0
Granule C2
Neuroblast C4
Granule C3
Granule
Neuroblast

Figure S16. Results of SCASL applied on 10X data of Dentate gyrus cells.

(A) TSNE plot showing clustering of 2442 cells by SCASL based on the AS landscapes. The cells are color labeled by the clusters defined by SCASL (left) and cell types defined by marker genes (right). The principal component analysis (PCA) is performed with a dimensionality reduction number of 20.

(B) The dot plot shows the expression of marker and functional genes in poor differentiation (Neuroblast) and well differentiation (Granule cell). The dot sizes represent the proportions of cells with expression, and the color scale represents the mean expression level.

(C) TSNE plot showing clustering of 2442 cells by Seurat based on the gene expression profiles with 3000 variable features. The cells are color labeled by the clusters defined by SCASL (left) and cell types defined by marker genes (right). The principal component analysis (PCA) is performed with a dimensionality reduction number of 20.
