# Supplementary material for: Interrogations of single-cell RNA splicing landscapes with SCASL define new cell identities with physiological relevance
Source: Nat Commun. 2024 Mar 9;15:2164. doi: 10.1038/s41467-024-46480-9 (PMC10925056; doi:10.1038/s41467-024-46480-9)
Supplement: Supplementary file 3 — Reporting Summary [file 41467_2024_46480_MOESM3_ESM.pdf]

nature portfolio

Corresponding author(s): Xuerui Yang

Last updated by author(s): Feb 20, 2024

Reporting Summary

Nature Portfolio wishes to improve the reproducibility of the work that we publish. This form provides structure for consistency and transparency in reporting. For further information on Nature Portfolio policies, see our Editorial Policies and the Editorial Policy Checklist.

Statistics

For all statistical analyses, confirm that the following items are present in the figure legend, table legend, main text, or Methods section.

n/a	Confirmed	
☐	☒	The exact sample size (*n*) for each experimental group/condition, given as a discrete number and unit of measurement
☐	☒	A statement on whether measurements were taken from distinct samples or whether the same sample was measured repeatedly
☐	☒	The statistical test(s) used AND whether they are one- or two-sided<br>*Only common tests should be described solely by name; describe more complex techniques in the Methods section.*
☒	☐	A description of all covariates tested
☐	☒	A description of any assumptions or corrections, such as tests of normality and adjustment for multiple comparisons
☐	☒	A full description of the statistical parameters including central tendency (e.g. means) or other basic estimates (e.g. regression coefficient) AND variation (e.g. standard deviation) or associated estimates of uncertainty (e.g. confidence intervals)
☐	☒	For null hypothesis testing, the test statistic (e.g. *F*, *t*, *r*) with confidence intervals, effect sizes, degrees of freedom and *P* value noted<br>*Give P values as exact values whenever suitable.*
☒	☐	For Bayesian analysis, information on the choice of priors and Markov chain Monte Carlo settings
☐	☒	For hierarchical and complex designs, identification of the appropriate level for tests and full reporting of outcomes
☐	☒	Estimates of effect sizes (e.g. Cohen's *d*, Pearson's *r*), indicating how they were calculated

Our web collection on statistics for biologists contains articles on many of the points above.

Software and code

Policy information about availability of computer code

Data collection	All raw fastq files of scRNA-seq data were downloaded from GEO database with fastq-dump software (v3.0.1)
Data analysis	All the scRNA-seq datasets we collected were pre-processed with SCASL (v1.0.0) developed by us, including extracting junction information to make matrix, quality filtering, making variable shear ratio matrix, imputation of missing values, retaining NA position information, et al. SCASL (v1.0.0) was used to identify novel single-cell clusters based on AS levels and reveal important differential splicing events. All source codes of SCALS were all deposited in the github (https://github.com/xryanglab/SCASL). Other softwares and tools we used in the present study include hisat2 v2.1.0, samtools v1.9, bamtools v2.5.1, leafcutter v0.2, R >=3.6, Rstudio and python >=3.6, et al.

For manuscripts utilizing custom algorithms or software that are central to the research but not yet described in published literature, software must be made available to editors and reviewers. We strongly encourage code deposition in a community repository (e.g. GitHub). See the Nature Portfolio guidelines for submitting code & software for further information.

Data

Policy information about availability of data

All manuscripts must include a data availability statement. This statement should provide the following information, where applicable:

- Accession codes, unique identifiers, or web links for publicly available datasets
- A description of any restrictions on data availability
- For clinical datasets or third party data, please ensure that the statement adheres to our policy

All single-cell data utilized in this manuscript were sourced from publicly available databases, with the exception of the T cell dataset, which was deposited in the

Gene Expression Omnibus (GEO). The accession numbers associated with the datasets are as follows: GSE123837, GSE118390, GSE90047, GSE71585, GSE84465 and GSE95315. The T cell data are available under restricted access for download restrictions on the China National Center for Bioinformation (CNCB) platform, access can be obtained by submitting an application to the Zhang Lab (please contact: hengsj@mail.cbi.pku.edu.cn), and can subsequently be downloaded from the national genomics data center with the accession number HRA000069. Source data are provided with this paper.

Research involving human participants, their data, or biological material

Policy information about studies with human participants or human data. See also policy information about sex, gender (identity/presentation), and sexual orientation and race, ethnicity and racism.

Reporting on sex and gender	There are no human research participants.
Reporting on race, ethnicity, or other socially relevant groupings	There are no human research participants.
Population characteristics	There are no human research participants.
Recruitment	There are no human research participants.
Ethics oversight	There are no human research participants.

Note that full information on the approval of the study protocol must also be provided in the manuscript.

Field-specific reporting

Please select the one below that is the best fit for your research. If you are not sure, read the appropriate sections before making your selection.

[x] Life sciences [ ] Behavioural & social sciences [ ] Ecological, evolutionary & environmental sciences

For a reference copy of the document with all sections, see nature.com/documents/nr-reporting-summary-flat.pdf

Life sciences study design

All studies must disclose on these points even when the disclosure is negative.

Sample size	We collected a total of seven sets of public single-cell sequencing data from humans and mice, and the cell types cover various cell types, including tumor cells, healthy tissue cells and immune cells. These datasets were simply used to test the performance of SCASL in cell clustering and further downstream analyses. The number of cells per data set varies from 400 to 4000 to confirm the broad range of applications of SCASL. No statistical method was used to predetermine sample size. No data were excluded from the analyses.
Data exclusions	No data was excluded from the analyses.
Replication	All single-cell sequencing data are derived from patient or mouse tissue with more than 400 cells per dataset, and each subpopulation in the clustering results has more than 10 cells. When all the data, environment and parameters are consistent, the clustering results are basically consistent.
Randomization	This manuscript does not address experimental validation.
Blinding	Blinding was not relevant as no direct comparison between experimental groups was done. The study is mainly analysis of the public data.

Reporting for specific materials, systems and methods

We require information from authors about some types of materials, experimental systems and methods used in many studies. Here, indicate whether each material, system or method listed is relevant to your study. If you are not sure if a list item applies to your research, read the appropriate section before selecting a response.

Materials & experimental systems

n/a	Involved in the study	
x		Antibodies
x		Eukaryotic cell lines
x		Palaeontology and archaeology
x		Animals and other organisms
x		Clinical data
x		Dual use research of concern
x		Plants

Methods

n/a	Involved in the study	
x		ChIP-seq
x		Flow cytometry
x		MRI-based neuroimaging
